# Is there a maximal effect of tranexamic acid in patients undergoing total knee arthroplasty? A randomized controlled trial

**DOI:** 10.1002/mco2.23

**Published:** 2020-08-27

**Authors:** Yiting Lei, Jinwei Xie, Qiang Huang, Fuxing Pei

**Affiliations:** ^1^ Department of Orthopedics The First Affiliated Hospital of Chongqing Medical University Chongqing People's Republic of China; ^2^ Department of Orthopedics West China Hospital Sichuan University Chengdu People's Republic of China

**Keywords:** blood loss, fibrinolysis, inflammation, total knee arthroplasty, tranexamic acid

## Abstract

The optimal dosing regimen of tranexamic acid (TXA) has not been determined in total knee arthroplasty (TKA). In this study, patients were randomized to receive a high initial‐dose (60 mg/kg) TXA before incision, followed by five doses 3, 6, 12, 18, and 24 hours later (A), or three doses 3, 12, and 24 hours later (B), or a single dose 3 hours later (C). The primary outcome was perioperative blood loss. Other outcomes such as, hemoglobin level, transfusion, the levels of fibrin (ogen) degradation products (FDP), D‐dimer, C‐reactive protein (CRP) and interleukin‐6 (IL‐6), coagulation parameters, and adverse events were also compared. The results showed that individuals in Groups A and B had reduced total and hidden blood loss (HBL), lower FDP, D‐dimer, CRP, and IL‐6 levels than in Group C. Such differences were also detected in HBL between Groups A and B. No differences were observed in other outcomes between Groups A and B. No differences were observed in coagulation parameters and adverse events among the three groups. In conclusion, a high initial‐dose (60 mg/kg) TXA before TKA followed by three doses can be sufficient to achieve maximal effects on total blood loss, fibrinolysis, and inflammation.

## Graphical abstract

We designed this randomized controlled trial to explore the optimal dosing regimen of tranexamic acid (TXA) in total knee arthroplasty (TKA), and suggested a high initial dose TXA followed by three doses of 1 g TXA 3, 12, and 24 hours later is sufficient to achieve maximal effects on blood loss, fibrinolysis, and inflammation in TKA.

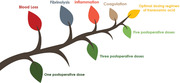

## 1 INTRODUCTION

Total knee arthroplasty (TKA) is viewed as the best surgical approach to treat severe knee osteoarthritis and other knee diseases.^1^ However, surgical trauma is always companied by significant hyperfibrinolysis, which can lead to considerable blood loss and rising demand for blood transfusions.^1^, ^2^ Although blood transfusion is a common procedure and generally safe,^3^ it still has a potential risk of morbidity and mortality.^4^ Therefore, various methods of blood conservation have been studied, including applying antifibrinolytic drugs.^1^, ^2^, ^5^

Tranexamic acid (TXA) is an antifibrinolytic drug that promotes a reduction in fibrinolysis.^2^ Numerous studies have demonstrated a reduction in perioperative bleeding in patients who received TXA.^1^, ^5^, ^6^, ^7^, ^8^ Nevertheless, the optimal dosage of TXA in TKA is still undetermined.^2^ It is reported that multiple‐dose regimen is superior to single‐dose regimen, and a preoperative dose followed by a postoperative dose could be the least amount of dosage to effectively reduce blood loss.^7^, ^9^, ^10^ Our preceding research has been conducted to compare different loading dosages, which proved the effectiveness of a high initial dose (60 mg/kg) TXA on blood loss, fibrinolysis, and inflammatory response.^11^ The results were encouraging, but it is still unclear whether the use of a high initial dose TXA can reduce the maintenance dosage, since a high loading dose tends to maintain an effective concentration for a longer period.

Therefore, the objective of our prospective, randomized, double‐blind trial was to explore the optimal dosing regimen of TXA in TKA. We hypothesized that with a high initial dose (60 mg/kg) TXA, less maintenance doses could be sufficient to achieve maximal effects on blood loss, fibrinolysis, and inflammation.

## 2 METHODS

### 2.1 Study design

The trial protocol was approved by the ethics committee (2017‐128) in accordance with the principles of the Helsinki Declarations. The trial has been registered prior to initiation (ChiCTR1800016640). All patients signed the written informed consent.

### 2.2 Patient cohort

From October 2018 to August 2019, we enrolled patients who had symptomatic, radiologically confirmed knee osteoarthritis and were scheduled for TKA in our medical center. Individuals were excluded if they had a history of pulmonary embolism (PE) or thromboembolic disease, an acquired or congenital coagulopathy, the current use of anticoagulant agents, renal or hepatic impairment, and allergy to TXA. Preoperative comorbidities were assessed after admission. Preoperative blood pressure was controlled below 140/90 mm Hg.

In total, there were 96 participants randomized to one of three groups. All patients received a high initial dose (60 mg/kg) TXA prior to operation. After the first administration, Group A received another five doses of 1 g TXA 3, 6, 12, 18, and 24 hours later; Group B received another three doses of 1 g TXA 3, 12, and 24 hours later; and Group C received another one dose of 2 g TXA 3 hours later.

### 2.3 Surgical procedures

All the surgical procedures were conducted by the same medical team under general anesthesia. Controlled hypotensive anesthesia (BP <100/60 mm Hg) was maintained during procedure. All the total knee prostheses were posterior stabilized cemented implants (PFC sigma). Drainage and tourniquet were not applied in any patients. To assess the volume of intraoperative blood loss (IBL), we meticulously recorded the weight of the gauze with blood. In addition, the amount of fluid collected in the aspirator was also recorded.

### 2.4 Postoperative care protocol

In our medical institution, all recruited patients were managed with the use of the same thromboprophylaxis protocol. Enoxaparin (Clexane; Sanofi‐Aventis, Paris, France), 2000 IU/day, was started 6 hours after TKA. Our protocol for routine discharge from the hospital was on the third day after surgery. After discharge, rivaroxaban (Xarelto, Bayer, Leverkusen, Germany) was given orally in a dose of 10 mg once daily for 10 days. Each patient was screened with clinical symptoms and physical examination to ensure no deep venous thrombosis was present. PE was investigated by clinical features. Patient with clinically suspected PE would receive a contrast‐enhanced chest computed tomography (CT) scan immediately. Doppler ultrasound was performed on the third postoperative day and repeated at 14 days and 90 days after surgery to detect venous thrombosis of lower extremities.

In our medical institution, the threshold for blood transfusion was as follows: (a) the hemoglobin level was equal to or less than 7 g/dL; (b) the hemoglobin level was in the range of 7‐10 g/dL, accompanied by intolerable symptom of anemia (rapid heartbeat, lightheadedness, polypnea, or reduced exercise tolerance).

### 2.5 Outcome assessments

The relevant demographic and operative details were collected for comparisons. The patient's blood volume,^12^ total blood loss (TBL),^13^ IBL,^14^ and hidden blood loss (HBL)^15^ were assessed as previously described.

Fibrinolysis factors were tested preoperatively and 1, 2, and 3 days after TKA. Inflammatory components, activated partial thromboplastin time (APTT), prothrombin time (PT), platelet count, and thrombelastograghy (TEG) were tested preoperatively and 1, 2, 3, and 14 days after TKA. TEG is a global assay of hemostasis, which has been shown to reflect the variation of coagulability.^16^, ^17^ In this randomized controlled trial (RCT), blood was extracted into a citrate tube and left at room temperature for at least 15 minutes before testing. TEG was assessed using a RapidTEG Reagent (Hemostasis system, Braintree, Chicago, IL).

The level of anti‐factor Xa activity (AFXa) is recommended to be monitored for dosage adjustment purposes in these patients receiving enoxaparin.^18^ In this RCT, blood was extracted into a CTAD tube, centrifuged at 2500 g for 18 minutes, and stored at −20 °C. Chromogenic substrate method was adopted for analysis of AFXa levels with the use of an Anti‐Xa assay kit (Shanghai Zhenyuan Diagnostic Supplies Technology Co.,Ltd., Shanghai, China). Enoxaparin was started 6 hours after TKA and continued once daily during the inpatient hospital stay. Based on the previous finding that the level of AFXa reached its peak at 4 hours after administration,^18^ we drew the blood one day before surgery, and repeated at 10 and 34 hours after TKA to assess AFXa. Transfusion rate and adverse events were recorded during hospitalization and 90‐day follow‐up period.

### 2.6 Data analysis

The sample size was calculated based on our pilot study with the use of PASS for Windows (version 11). It is shown that a sample size of 31 subjects per arm was required to detect a difference of 100 mL in TBL among groups with 90% power and *P* < .05. Anticipating an attrition rate of 22.5%, 120 patients had to be included.

All statistical analyses were performed with the use of SPSS for Windows (version 24). *P* values were judged significant if they were less than .05.

## 3 RESULTS

### 3.1 Baseline characteristics

From October 2018 to August 2019, there were 120 participants screened for participation in this trial. Among them, 13 were ineligible, 5 refused to consent, 4 dropped out, and 2 were lost to follow‐up. Eventually, 96 patients were involved in the final analysis. Baseline characteristics were similar among groups (Table 1).

**TABLE 1:** Demographic data of the patients receiving unilateral TKA

Demographic	Group A (n = 32)	Group B (n = 32)	Group C (n = 32)	*P* value
Age (year)	66.63 ± 6.70	65.25 ± 7.18	69.41 ± 7.49	.065
Gender (n)				.443
Male	3	4	7	
Female	29	28	25	
Height (cm)	157.53 ± 4.23	154.78 ± 6.71	157.47 ± 7.49	.143
Weight (kg)	64.50 ± 9.82	61.25 ± 7.91	63.47 ± 8.75	.329
BMI (kg/m^2^)	25.95 ± 3.60	25.55 ± 2.83	25.56 ± 2.84	.839
Hypertension (n)	14	14	19	.353
Diabetes (n)	4	1	5	.331
ASA class				.101
2 (n)	19	26	19	
3 (n)	13	6	13	
HSS score	47.50 ± 6.13	46.91 ± 6.55	45.81 ± 6.46	.564
ROM (°)	99.84 ± 17.53	95.47 ± 11.66	97.66 ± 17.04	.537
Operated side				.272
Left (n)	17	11	16	
Right (n)	15	21	16	
PBV (mL)	3750.09 ± 426.57	3550.20 ± 424.19	3695.11 ± 450.59	.169
Operation time (min)	72.38 ± 14.78	72.19 ± 15.56	77.53 ± 14.48	.273
INFV (mL)	410.94 ± 118.96	434.38 ± 151.04	453.13 ± 162.61	.511

Abbreviations: ASA, American Society of Anesthesiologists; BMI, body mass index; HSS, hospital for surgery; INFV, intraoperative intravenous fluid volume; PBV, patient blood volume; ROM, range of motion.

### 3.2 Perioperative blood loss and transfusion

The TBL, HBL, and maximum hemoglobin (Hb) drop in Groups A (*P *< .001, < .001, < .001) and B (*P *< .001, < .001, *P* = .007) were significantly lower than those in Group C. Such differences were also detected in HBL between Groups A and B (*P *= .049). However, no differences were found between Groups A and B in terms of TBL and maximum Hb drop (*P *= .068, .165). Furthermore, there were no significant differences in IBL and transfusion rate among the three groups (Table 2).

**TABLE 2:** Postoperative blood loss and transfusion

	Group A (n = 32)	Group B (n = 32)	Group C (n = 32)	*P* value	*P*1	*P*2	*P*3
TBL (mL)	478.60 ± 169.43	583.97 ± 162.87	862.82 ± 222.97	.000	.068	.000	.000
IBL (mL)	77.81 ± 19.13	71.88 ± 17.12	79.06 ± 16.14	.220	.368	.956	.233
HBL (mL)	400.79 ± 171.69	512.10 ± 157.64	783.76 ± 222.97	.000	.049	.000	.000
Max Hb drop (g/L)	17.63 ± 7.15	22.13 ± 5.84	29.72 ± 14.29	.000	.165	.000	.007
Transfusion (n)	0	0	1	1.000	1.000	1.000	1.000

*P*, *P* value of group A vs B vs C; *P*1, *P* value of group A vs B; *P*2, *P* value of group A vs C; *P*3, *P* value of group B vs C.

Abbreviations: HBL, hidden blood loss; IBL, intraoperative blood loss; Max Hb drop, maximum hemoglobin drop; TBL, total blood loss.

### 3.3 Fibrinolysis parameters

**FIGURE 1:**
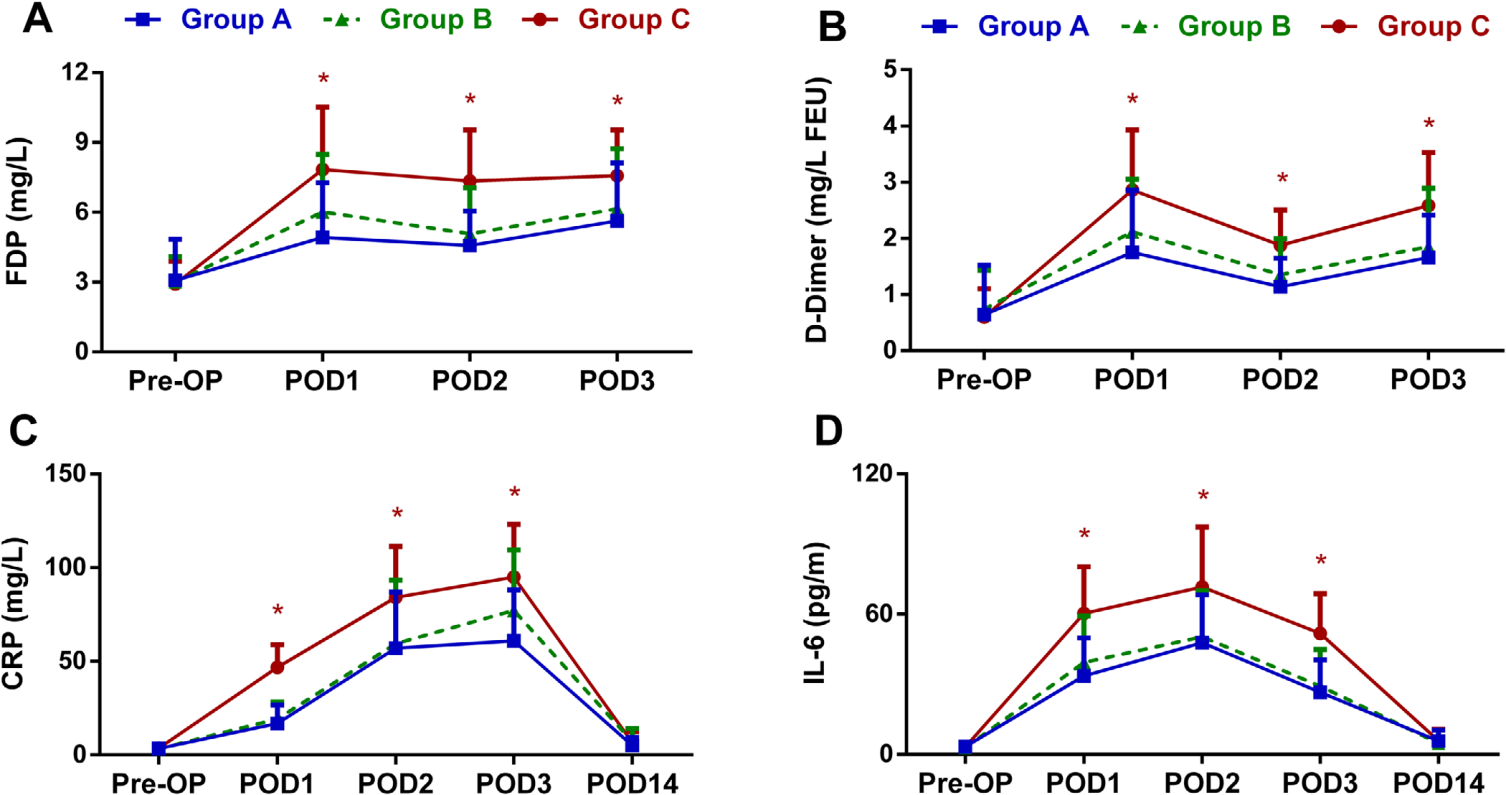
The perioperative levels of FDP (A), D‐dimer (B), CRP (C), and IL‐6 (D) in patients undergoing TKA. The levels of FDP and D‐dimer were reported to be able to reflect the level of fibrinolytic activity. CRP and IL‐6 are commonly used as biochemical markers of inflammation following TKA. *means *P *< .05. Abbreviations: CRP, C‐reactive protein; FDP, fibrin (ogen) degradation products; IL‐6, interleukin‐6; POD, postoperative; Pre‐OP, preoperative

As shown in Figure 1A,B, the levels of FDP on POD 1, 2, and 3 were significantly lower in Groups A (*P *= .007, .009, *P* < .001) and B (*P *= .030, .046, .023) compared to Group C. In addition, Groups A (*P *= .001, .002, *P* < .001) and B (*P *= .003, .002, .007) had significantly lower D‐dimer levels than Group C on POD 1, 2, and 3. The levels of FDP and D‐dimer were similar between Groups A and B.

### 3.4 Inflammation markers

As shown in Figure 1C,D, significantly lower C‐reactive protein (CRP) levels were detected in Groups A (*P *= .002, .006, *P* < .001) and B (*P *= .016, .034, .009) on POD 1, 2, and 3 compared to Group C. In addition, the interleukin‐6 (IL‐6) levels in Groups A (*P *= .007, .004, .023) and B (*P *= .029, .009, .037) were significantly lower than those in Group C on POD 1, 2, and 3. The levels of CRP and IL‐6 were similar between Groups A and B.

### 3.5 Coagulation parameters and complications

**FIGURE 2:**
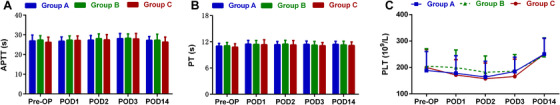
The perioperative levels of APTT (A), PT (B), and platelet count (C) in patients undergoing TKA. Abbreviations: APTT, activated partial thromboplastin time; PLT, platelet count; PT, prothrombin time; POD, postoperative; Pre‐OP, preoperative

**FIGURE 3:**
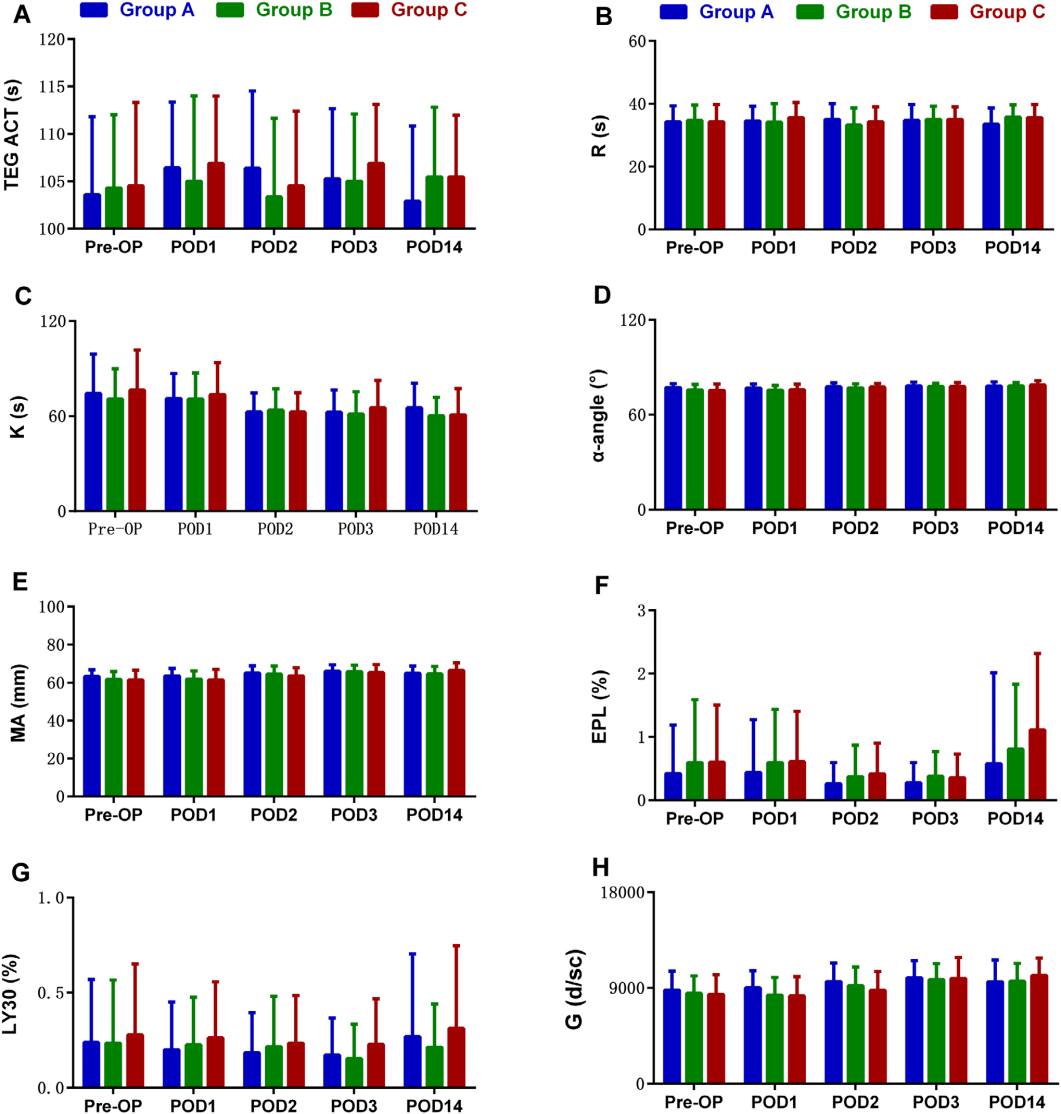
Perioperative TEG analysis in patients undergoing TKA. TEG is a global assay of hemostasis which has been shown to reflect the variation of coagulability. TEG parameters mainly include TEG ACT (A), R(B), K(C), α‐angle (D), MA (E), EPL (F), LY30 (G), and G (H). TEG ACT, thromboelastography‐generated activated clotting time; R, perioperative reaction time; K, kinetics; α‐angle, alpha‐angle; MA, maximum amplitude; EPL, estimate percent lysis; LY30, lysis rate at 30 minutes; Pre‐OP, preoperative; POD, postoperative

No differences were found among the three groups in terms of postoperative APTT, PT, platelet count, r‐TEG results, or AFXa (Figures 2 and 3, Table 3). Furthermore, the results of APTT, PT, platelet count, and r‐TEG were within the normal range, and the results of AFXa were within the recommended prophylactic range. No significant differences were observed in thrombotic events among the three groups within 90 days (Table 3). No treatment‐related complications such as PE, seizure, or cerebrovascular events occurred during hospitalization or 90‐day follow‐up period.

**TABLE 3:** Perioperative antifactor Xa level and postoperative thrombotic events

	Group A (n = 32)	Group B (n = 32)	Group C (n = 32)	*P* value
Anti‐factor Xa level (IU/mL)				
Pre‐OP	0.02 ± 0.03	0.01 ± 0.02	0.01 ± 0.02	.743
10 h PO	0.24 ± 0.07	0.20 ± 0.08	0.21 ± 0.08	.106
34 h PO	0.22 ± 0.08	0.22 ± 0.08	0.22 ± 0.08	.910
DVT				
POD 3 (n)	0	0	0	–
POD 14 (n)	0	1	0	1.000
POD 90 (n)	0	0	1	1.000
IVT				
POD 3 (n)	1	3	3	.693
POD 14 (n)	0	1	2	.771
POD 90 (n)	0	1	1	1.000

Abbreviations: DVT, deep venous thrombosis; IVT, intramuscular venous thrombosis; POD 3, postoperative day 3; POD 14, postoperative day 14; POD 90, postoperative day 90; Pre‐OP, preoperative; 10 h PO, 10 hours postoperatively; 34 h PO, 34 hours postoperatively.

## 4 DISCUSSION

TKA is known to be an efficacious treatment modality for severe knee arthrosis.^1^ However, surgical trauma that induces both the coagulation cascade and fibrinolysis can lead to significant blood loss.^2^ Accordingly, the focus of recent clinical studies has involved the use of antifibrinolytics.^1^, ^19^ TXA is an antifibrinolytic drug that can not only noncompetitively inhibit plasmin but also competitively inhibit the lysine binding site.^20^ Although numerous studies have proved the efficacy of TXA,^1^, ^21^, ^22^ the reports regarding the optimal protocol of TXA in TKA are conflicting.^1^, ^6^ As a cascade process, fibrinolytic activation is most easily controlled at its early phase.^23^ Therefore, the preoperative dose has been regarded as the most crucial dose.^24^, ^25^ It has been suggested that TXA at doses more than 61 mg/kg may raise the risk of seizure,^26^, ^27^ indicating that a dosage of 60 mg/kg might be the maximum safe dose. Our published study has demonstrated the superior efficacy of a high initial dose TXA (60 mg/kg) followed by five postoperative doses.^11^ However, the optimal postoperative TXA regimen still remains unclear. It is reported that it could be beneficial to maintain antifibrinolytic treatment in the postoperative period, and at least two perioperative doses could be required to be effective.^7^, ^9^, ^10^ Hourlier et al indicated that a higher loading dose could maintain an effective concentration for a longer period,^28^ which may explain why the previous works failed to detect the efficacy of single low‐dose administration. Based on the above studies, we speculate that a high initial dose (60 mg/kg) TXA could reduce the volume of usage and the frequency of drug administration after TKA.

To our knowledge, this is the first attempt to explore the optimum postoperative TXA regimen to maximize the hemostatic effects and minimize the side effects with a high initial dose (60 mg/kg) TXA. With the same preoperative protocol, patients in our study were treated with another five doses of TXA (six‐dose regimen), or another three doses of TXA (four‐dose regimen), or another one dose of TXA (two‐dose regimen). The main finding of this present study is that four‐dose regimen can be sufficient to achieve a maximal effect on fibrinolysis, which could play a role in reducing TKA‐associated blood loss. The level of FDP and D‐dimer was tested to evaluate fibrinolysis. Individuals in two‐dose group had higher FDP and D‐dimer levels than those in four‐dose and six‐dose groups. Besides, the four‐dose and six‐dose regimens significantly reduced TBL, HBL, and maximum hemoglobin drop as compared with the two‐dose regimen. Since fibrinolysis remains at a high level for 18‐24 hours after TKA,^29^, ^30^ repeated doses up to 24 hours could be more effective in inhibiting postoperative fibrinolysis, which could explain the differences between the two‐dose regimen and the four‐ and six‐dose regimens. Interestingly, no differences were observed between the four‐ and six‐dose regimens in terms of blood‐saving and antifibrinolytic efficacy. The results confirmed our hypothesis that a high loading dose could prolong the maintenance dosage intervals and reduce postoperative dosage.

Surgical stress elicited by TKA may increase the release of inflammatory components^31^ and may have negative influence on postoperative recovery.^32^ Despite various studies proving the efficacy of TXA with single or multiple boluses on fibrinolysis, no consensus has been reached on its anti‐inflammatory properties.^1^, ^5^, ^33^ Previous studies showed that plasminogen could bind to inflammatory cells like monocytes, macrophages, and neutrophils, and D‐dimer could raise the level of biologically active cytokines.^34^, ^35^ Thus, it is logical to assume that there might be a cross‐talk between fibrinolysis and inflammation, and TXA may have an anti‐inflammatory effect through its inhibition of plasmin formation.^33^, ^36^, ^37^ In this present study, the level of CRP and IL‐6 was measured to show the inflammatory status.^38^ We found significantly higher concentrations of CRP and IL‐6 in the two‐dose group than in the four‐ and six‐dose groups. As expected, no difference was found between the four‐ and six‐dose regimens, which was in accordance with difference in fibrinolysis parameters. Our results might provide an indirect support for the correlation between TXA and inflammation, and suggest that four‐dose regimen can be sufficient to achieve a maximal effect on anti‐inflammation.

It has been reported that the effect of anticoagulant therapy might be weakened by some hemostatic agents.^39^, ^40^, ^41^ Nevertheless, relatively little knowledge is available related to the correlation between TXA and anticoagulant drug. Honda et al and Levy et al used PT to assess the effect of TXA on edoxaban and rivaroxaban, and found that TXA failed to decrease the effects of anticoagulant therapy.^42^, ^43^ However, the results of PT assays could be influenced by some extraneous factors, and anti‐Xa assay could be less impacted by variability.^44^, ^45^ To our knowledge, this is the first study based on AFXa, APTT, and PT to assess the influence of different postoperative TXA regimens with a high initial dose TXA on the effects of enoxaparin. No significant differences were observed among the three groups in terms of AFXa, APTT, and PT, and the results of APTT and PT remained within their normal ranges. More importantly, the postoperative AFXa remained within the recommended prophylactic range (0.1–0.3 IU/mL),^46^ indicating that a high loading dose of TXA followed by different maintenance dosage has no influence on the anticoagulant effect.

Although previous studies on TXA and thrombosis failed to show any thrombogenic effect, little focus has been shifted to the safety of different postoperative TXA regimens with a high initial dose TXA.^1^, ^6^, ^47^, ^48^, ^49^ Besides, the majority of previous RCTs were conducted with conditions of relatively small sample size,^1^, ^2^, ^5^ and it is known that at least 2000 individuals per group would be required to find a difference in this low‐incidence event.^50^ In addition to clinical features, we also used TEG, PT, APTT, and platelet count to evaluate the effect of TXA on coagulation and platelet function in this present study. No differences in these parameters were observed among the three groups, and all of them were in their normal ranges, providing a further confirmation with regard to the safety of TXA.

Several limitations should be noted when interpreting the results. First, the sample size of this present trial may not large enough to detect potential complications related to TXA. Besides, the study's generalizability to surgical populations is limited, since we excluded the patients with preexisting coagulopathy. Last but not the least, this study mainly compared the hemostatic, antifibrinolytic, and anti‐inflammatory effects of different postoperative TXA regimens, the effects on postoperative pain level and knee function were absent in this trial, which merit further investigation. Despite its limitations, it is a prospective RCT with consistent anesthesia protocol, surgical procedures, and perioperative care regimens.

## 5 CONCLUSION

In conclusion, a high initial dose (60 mg/kg) followed by three doses of 1 g TXA 3, 12, and 24 hours later has favorable effects on TBL, fibrinolysis, and inflammation in TKA. However, although this study suggested an optimal dosing regimen among the three regimens, there might exist a better regimen with different initial dose and maintenance doses. Further studies in large scales are still required to evaluate the benefit to risk ratio before final recommendations.

## CONFLICT OF INTEREST

The authors declare no conflict of interest.

## FUNDING INFORMATION

National Health and Family Planning Commission of the People's Republic of China (CN) program; Grant Number: 201302007.

## ETHICAL APPROVAL

This trial was approved by the institutional review board (2017‐128).

## INFORMED CONSENT

Informed consent was obtained from all individual participants included in the study.
